# C mobilisation in disturbed tropical peat swamps: old DOC can fuel the fluvial efflux of old carbon dioxide, but site recovery can occur

**DOI:** 10.1038/s41598-019-46534-9

**Published:** 2019-08-07

**Authors:** Susan Waldron, Leena Vihermaa, Stephanie Evers, Mark H. Garnett, Jason Newton, Andrew C. G. Henderson

**Affiliations:** 1 School of Geographical and Earth Sciences, University of Glasgow, Glasgow, G12 8QQ UK; 2 School of Natural Sciences and Psychology, Liverpool John Moores University, Liverpool, UK; 3 School of Geographical and Environmental Sciences, University of Nottingham Malaysia Campus, Kuala Lumpur, Malaysia; 4 NERC Radiocarbon Facility, Scottish Enterprise Technology Park, East Kilbride, G75 0QF UK; 5 Scottish Universities Environmental Research Centre, Scottish Enterprise Technology Park, East Kilbride, G75 0QF UK; 6 School of Geography, Politics & Sociology, Newcastle University, Newcastle-upon-Tyne, NE1 7RU UK

**Keywords:** Carbon cycle, Environmental monitoring, Environmental impact

## Abstract

Southeast-Asian peat swamp forests have been significantly logged and converted to plantation. Recently, to mitigate land degradation and C losses, some areas have been left to regenerate. Understanding how such complex land use change affects greenhouse gas emissions is essential for modelling climate feedbacks and supporting land management decisions. We carried out field research in a Malaysian swamp forest and an oil palm plantation to understand how clear-felling, drainage, and illegal and authorized conversion to oil palm impacted the C cycle, and how the C cycle may change if such logging and conversion stopped. We found that both the swamp forest and the plantation emit centuries-old CO_2_ from their drainage systems in the managed areas, releasing sequestered C to the atmosphere. Oil palm plantations are an iconic symbol of tropical peatland degradation, but CO_2_ efflux from the recently-burnt, cleared swamp forest was as old as from the oil palm plantation. However, in the swamp forest site, where logging had ceased approximately 30 years ago, the age of the CO_2_ efflux was modern, indicating recovery of the system can occur. ^14^C dating of the C pool acted as a tracer of recovery as well as degradation and offers a new tool to assess efficacy of restoration management. Methane was present in many sites, and in higher concentrations in slow-flowing anoxic systems as degassing mechanisms are not strong. Methane loading in freshwaters is rarely considered, but this may be an important C pool in restored drainage channels and should be considered in C budgets and losses.

## Introduction

As atmospheric CO_2_ concentrations continue to rise, understanding how carbon is lost from terrestrial stores remains crucial. Drainage systems are important conduits for terrestrial C export as dissolved and particulate organic carbon (DOC, POC respectively) and dissolved inorganic carbon (DIC) (e.g.^1–3^). Some terrestrial loss is exported to marine systems where it can be degraded in the water column or sequestered^4^. However, some fluvial C load is, or can become, CO_2_, and will be degassed to the atmosphere^5^, bypassing storage. Insensitive land use change may drive more terrestrial C export, and if not sequestered elsewhere, CO_2_ may be degassed – sourced either from the inorganic C pool or from reprocessing of organic C. To understand and model these processes in a future C cycle we need to identify when land use change mobilises older stores of terrestrial C which were previously largely unavailable: release of this C to the atmosphere would be a climate warming feedback. In short when do ‘older and slower’ C cycles becomes ‘shorter and faster’? ^14^C dating can help here.

The ^14^C age of fluvial C export is generally young^6,7^ indicating that recently-fixed C dominates export. However, C export that is hundreds to thousands of years old indicates that older C stores contribute to this source, for example through drainage of land for oil palm plantation^8^. Thus, disturbed landscapes that degas old CO_2_ from catchment drainage can be identified to directly exert a positive feedback to global warming. However, whether old C is degassed has had little focus in landscapes subject to large-scale human-induced land use change.

Large-scale land use change causing loss of important terrestrial C stores is occurring in tropical peatlands. These have been under considerable pressure for redevelopment for palm oil production, pulp and paper plantations and to facilitate timber harvesting through drainage^9,10^. These land use changes can cause secondary disturbances that further increase C loss, such as increasing fire susceptibility from over-draining. Thus, the history of disturbance can be multi-layered and although current management may be to secure C stores, past land use may leave a legacy – but this too has been little explored.

**Figure 1:**
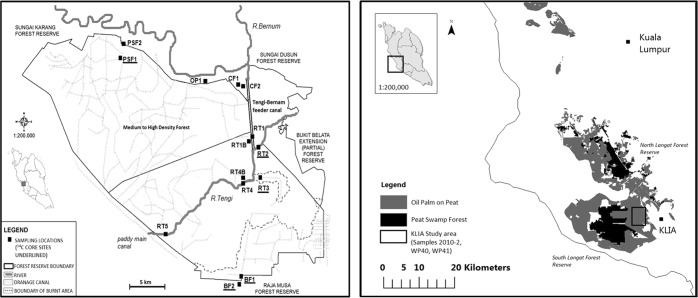
The North Selangor Peat Swamp Forest (left, termed ‘forest’) annotated with the sampling locations, and the location of the oil palm plantation (termed ‘OP-plantation’) in south Selangor within the vicinity of Kuala Lumpur International airport (KLIA) and South Langat Forest Reserve.

We studied two disturbed tropical peatland sites to explore how fluvial CO_2_ efflux reveals the ‘state’ of the terrestrial-aquatic-atmospheric C cycle - a feedback to global warming, or a system in recovery? Our field sites in Selangor state, peninsular Malaysia (Fig. 1) were the North Selangor Peat Swamp Forest (NSPSF), and an established oil palm plantation in south Selangor within the vicinity of Kuala Lumpur International airport and South Langat Forest Reserve (KLIA). We chose these sites because of complex land use history and the proximity of different land use pressures allowing sampling of both sites in short succession. We hypothesised that (i) CO_2_ degassed from fluvial systems draining peatlands would be old in sites subject to conversion or logging, but (ii) if a site was being successfully managed to support recovery of a more natural ecosystem, a modern C cycle would be prevalent. Radiocarbon dating of key C pools was used to test these hypotheses. Hydrochemical profiling, including assessing whether surface waters contained methane, supported deeper understanding of the local C cycle. To explore how land use changes influenced C export from on-going natural processes, we utilised Bayesian modelling of ^14^C ages to identify the credible contribution intervals of differently-aged carbon reservoirs.

## Results

We present here data from drainage in the two disturbed peatlands sites: a tropical swamp forest (NSPSF), and to the south of this, an oil palm plantation. With the swamp forest, our sampling sites had varying degrees of disturbance, from recovering peatland forest to logged forest. The oil palm plantation had no restored areas. The sample site description, location and sampling date, water chemistry, gas efflux rates and fluvial carbon concentrations and isotopic signatures, for all samples sites are given in the Supplementary Material (Tables SI.1 and SI.2); Table 1 and 2 detail the ‘primary’ samples which have ^14^C data. For simplicity where possible we refer to NSPSF as ‘forest’ and KLIA as ‘OP-plantation’, but there are times individual sites need to be named. Although the samples were collected over a 5-day period, there was no rain and so differences in water chemistry are not due to changes that can occur during high rainfall (e.g.^11^).

**Table 1:** Water chemistry and C determinants for key samples.

Location	Area	Site	pH	SC	%DO	Temp°C	[Ca]	[DIC]	δ^13^C-DIC	[DOC]	[POC]	CO_2_ efflux	[CH_4_-aq]
**Peat swamp forest logged**													
RT2	NSPSF	Tengi River	6.30	92.8	26.1	27.8	1.95	7.32	−9.3	11.7	2.54	1.60 ± 0.07	2.5
RT3	NSPSF	Tengi tributary	3.90	45.0	64.0	29.3	0.49	2.52	−23.4	45.9	1.79	1.78 ± 0.42	6.6
BF1	NSPSF	Illicitly cleared	3.74	75.2	6.7	29.7	0.96	5.40	−25.0	83.6	0.94	2.00 ± 0.39	15.3
BF2	NSPSF	Illicitly cleared	3.71	74.7	19.3	28.9	0.95	6.00	−25.7	83.0	0.76	23.99 ± 3.4	3.5
**Peat swamp forest logging moratorium**													
PSF1	NSPSF	Unlogged	3.64	129.7	1.6	25.6	0.93	4.92	−23.8	144.8	4.35	1.3 ± 0.04*	247.5
**Oil Palm Plantation**													
WP40	KLIA	Canal drain	3.88	334.3	25.5	30.0	6.90	3.12	−18.6	22.5	11.52	0.69 ± 0.11*	0.5
WP41	KLIA	Canal drain	4.06	206.6	54.6	27.7	2.91	0.84	−24.5	109.0	12.07	0.51 ± 0.08*	1.4

[DIC], [DOC], [POC] and [Ca] are in mg/l C, δ^13^C-DIC is in ‰, CO_2_ efflux is μmol C/m^2^/sec, [CH_4_-C_aq_] is μg/l, specific conductivity (SC) in μS/cm. Efflux at BF2 were taken in areas of turbulent and smoother water and thus shows considerable ranges.

^*^Here the chamber had to be gently agitated to break the boundary layer and facilitate CO_2_ efflux, so this is a maximum efflux rate under these conditions and more representative of efflux with a gentle breeze.

### Water chemistry

Most of the primary samples (Table 1) had water chemistry strongly influenced by organic soils (pH < 4, SC < 300 μS/cm, high [DOC], δ^13^C-DIC more typical of the C3 vegetation source, median of −23.8‰, and [DIC] < 7.3 mg/l C). The regional drainage in the forest (RT2, the River Tengi, Fig. 1) had chemistry more typical of contact with rock i.e. with a groundwater contribution. Here the pH, SC and [Ca] are greater, [DOC] is less, δ^13^C_DIC_ is more ^13^C-enriched and [DIC] is greater. The OP-plantation samples had greater SC and [Ca] than typical of peat drainage (e.g.^12^), particularly WP40 which had the lower [DOC]. Only in forest regional drainage (e.g. RT2) was [Ca] greater than peaty sites (e.g. RT1B, Table SI3), reflecting more groundwater influence. The higher SC at OP-plantation may come from liming and/or other herbicides and fertilisers. Alternatively, a non-peat, higher conductivity water may have diluted the DOC pool. Observed water management includes opening sluice gates during low water, to allow water in from nearby non-peat plantation main drains, which can be tracked back through housing and industrial zone areas. Such inflow could be influencing C dynamics, accommodated by including a ^14^C-dead C source in SIAR modelling.

As with many freshwater systems, the DOC pool was significantly larger than the DIC or POC pool. δ^13^C_DOC_ showed site-specific differences, with internally consistent and more-depleted δ^13^C_DOC_ in the forest (−29.4 ± 0.2‰, n = 4, Table 2) than OP-plantation (−27.5, −28.5‰). However, except for forest-RT1 and forest-BF2 (efflux of 18.23 and 3.58 μmol C/m^2^/sec respectively, Table SI3), CO_2_ efflux rates were similar between forest and OP-plantation, ranging from 0.51 to 2.00 μmol C/m^2^/sec. δ^13^C of the effluxed CO_2_ was either comparable or more ^13^C-enriched (Table 2) than the DIC pool (Table 1) in all cases except for forest-RT2, where it was significantly less ^13^C-enriched. Forest-RT2 is the only site where the pH was sufficiently high that the DIC pool would comprise CO_2(aq)_ and bicarbonate, and the free CO_2_ efflux (−17.4‰) could be more ^13^C-depleted than the DIC pool (−9.3‰) alone due to inter-isotope fractionation (e.g.^11^).

**Table 2:** ^14^C (% enrichment) and δ^13^C (‰) for paired CO_2_ efflux–DOC and peat samples collected to identify the impact of different land use.

Location	Site type		Lab code	CO_2_ % Modern ± 1σ	CO_2_^14^C Age BP ± 1σ	δ^13^C-CO_2_	Lab code	DOC % Modern ± 1σ	DOC ^14^C Age BP ± 1σ	δ^13^C-DOM
Location	Area	Depth (cm)	Lab code	Upper peat % Modern ± 1σ	Surface peat Age BP	δ^13^C-peat	Lab code	Lower peat % Modern ± 1σ	Basal peat Age BP ± 1σ	δ^13^C-peat
RT2	Forest		SUERC-47910	96.22 ± 0.44	310 ± 37	−17.4	SUERC-49328	96.65 ± 0.44	273 ± 37	−29.4
RT3	Forest		—	—	—	—	SUERC-49327	98.48 ± 0.45	123 ± 37	−29.4
BF1	Forest		SUERC-47911	93.07 ± 0.41	577 ± 35	−24.1	SUERC-49329	94.17 ± 0.41	483 ± 35	−29.6
BF2	Forest		SUERC-47912	93.01 ± 0.43	582 ± 37	−25.5	SUERC-49330	94.03 ± 0.41	495 ± 35	−29.0
PSF1	Forest (log halt)		SUERC-47913	102.86 ± 0.45	After 1950AD	−23.6	SUERC-49331	99.96 ± 0.46	3 ± 37	−29.7
WP40	OP-Plantation		SUERC-47914	96.92 ± 0.42	251 ± 35	−16.2	SUERC-49334	91.71 ± 0.42	695 ± 37	−27.5
WP41	OP-Plantation		SUERC-47915	93.18 ± 0.43	567 ± 37	−14.5	SUERC-49335	92.93 ± 0.40	589 ± 37	−28.5
WP40	OP-Plantation	3–8	SUERC-72693	94.73 ± 0.34	435 ± 29 (3–8 cm)	−30.1	SUERC-65199	72.87 ± 0.27	2542 ± 30 (181–183 cm)	−27.2
2010–2	OP-Plantation	3–6	SUERC-72694	91.77 ± 0.33	690 ± 29 (3–6 cm)	−29.5	SUERC-65204	73.49 ± 0.25	2474 ± 28 (36–38 cm)	−27.0
PSF2* (Close to PSF1)	Forest	10–13	SUERC-75684	102.12 ± 0.47	After 1950AD (10–13 cm)	−31.5	SUERC-75686	61.84 ± 0.29	3861 ± 37 (90–100 cm)	−29.7
MOP2* (Close to BF1/BF2)	Forest	—	—	—	—	—	SUERC-75693	61.19 ± 0.28	3946 ± 37 (70–80 cm)	−29.5

**Figure 2:**
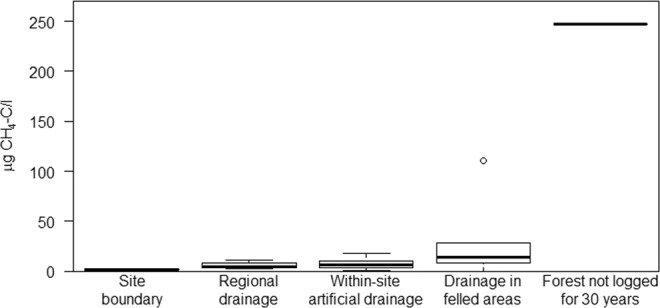
CH_4_ concentrations as a function of land use across the forest (NSPSF) and oil palm plantation site (KLIA). All data have been pooled for this land use consideration (see Table SI4 for individual site categorization). The graph shows the maximum, minimum, median and 1^st^ and 3^rd^ quartiles of the different categories. Note the forest not logged for 30 years has only one sample.

CH_4_ concentrations in all disturbed forest and OP-plantation sites and the main drainage channel were low: 0.5–15.3 µg CH_4_-C/l (Fig. 2, Table 1). The forest and the OP-plantation sites had comparable CH_4_ concentrations (Fig. SI1), but when considered as a function of land use across sites, greater and more variable concentrations were noted in clear-felled sites e.g. not detectable − 110.8 µg CH_4_-C/l (Fig. 2, Tables SI3, SI4). However, the [CH_4_] in the forest site unlogged for years (PSF1) was considerably higher and significantly different to all other land use classes: 247.5 µg CH_4_-C/l., (p-value < 0.001 in a F-test, when the results were tested with the least significant difference test using Bonferroni correction for multiple comparisons). DO was undersaturated at all sites (1.6–80.8%) and in some places, the water can be considered anoxic (forest: BF1; PSF1). Sites with the lower %DO tend to have the higher CH_4_ concentrations, but not always e.g. forest-CF2 (Table SI3).

**Figure 3:**
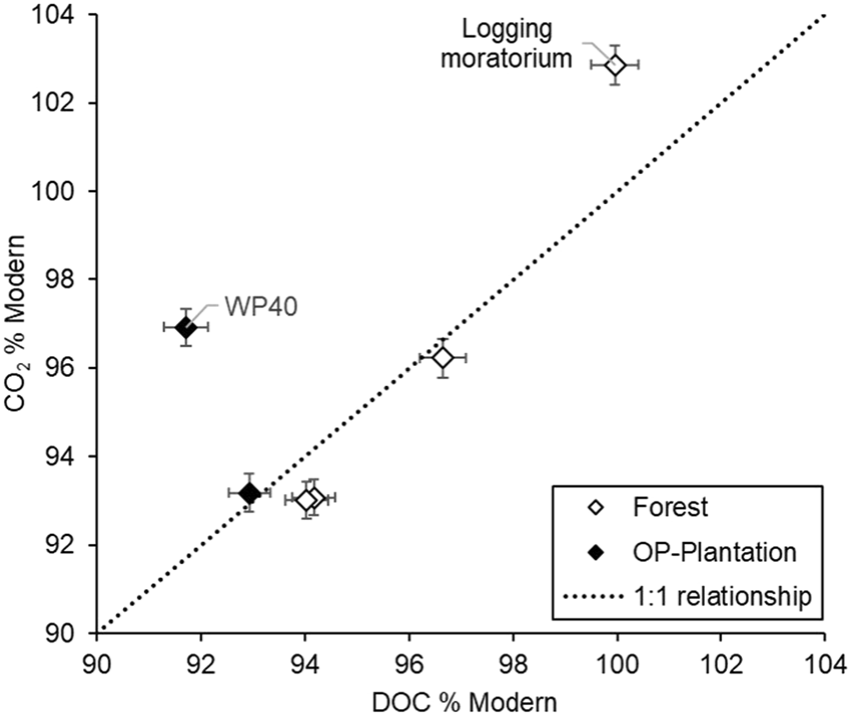
Relationship between ^14^C of fluvial CO_2_ efflux and of DOC for samples from the forest reserve and the OP-plantation.

The ^14^C content of the CO_2_ efflux ranged from 93.01 to 102.86% Modern (Table 2), which is the equivalent age of a net gas efflux ranging from 582 years BP to “modern” (i.e. <~60 years old). Two forest sites with similar CO_2_ and similar DOC ages are on the same drainage channel (BF1 and BF2, upstream and downstream of a weir). The youngest CO_2_ came from forest-PSF1, in the area that had not been logged for ~30 years. The ages of two samples from the OP-plantation differed by almost 300 years, although the sampling locations were less than 1 km apart. At all sites, the DOC was also pre-modern, ranging from 91.71 to 99.96% Modern, which is the equivalent of a DOC pool ranging from 695 to 3 years BP. At OP-plantation WP40 the DOC was considerably older than the CO_2_ efflux. At forest-PSF1 the CO_2_ efflux was likely composed of slightly younger C than the DOC, with the former having a clear post-bomb ^14^C concentration, and the latter a %modern range that spans the pre- and post-bomb periods. DOC and CO_2_ evasion are of similar age (within 100 years) at all other sampling points (Fig. 3).

The top and basal ages of the OP-plantation peat cores collected were respectively 435 ± 29/2542 ± 30 BP (WP40) and 690 ± 29/2474 ± 28 BP (KLIA 2010) (Table 2). Thus, it appears peat formation initiated in this area approximately 2550 years ago. Our basal peat age for the forest very close to PSF1 was 3861BP; the corresponding surface sample was modern in age. Basal peat sampled close to forest-BF1 and -BF2 was of similar age at 3946 BP. Thus, peat formation in the North Selangor Peat Swamp forest site occurred approximately 1300 years earlier.

### SIAR Mixing model outputs

**Figure 4:**
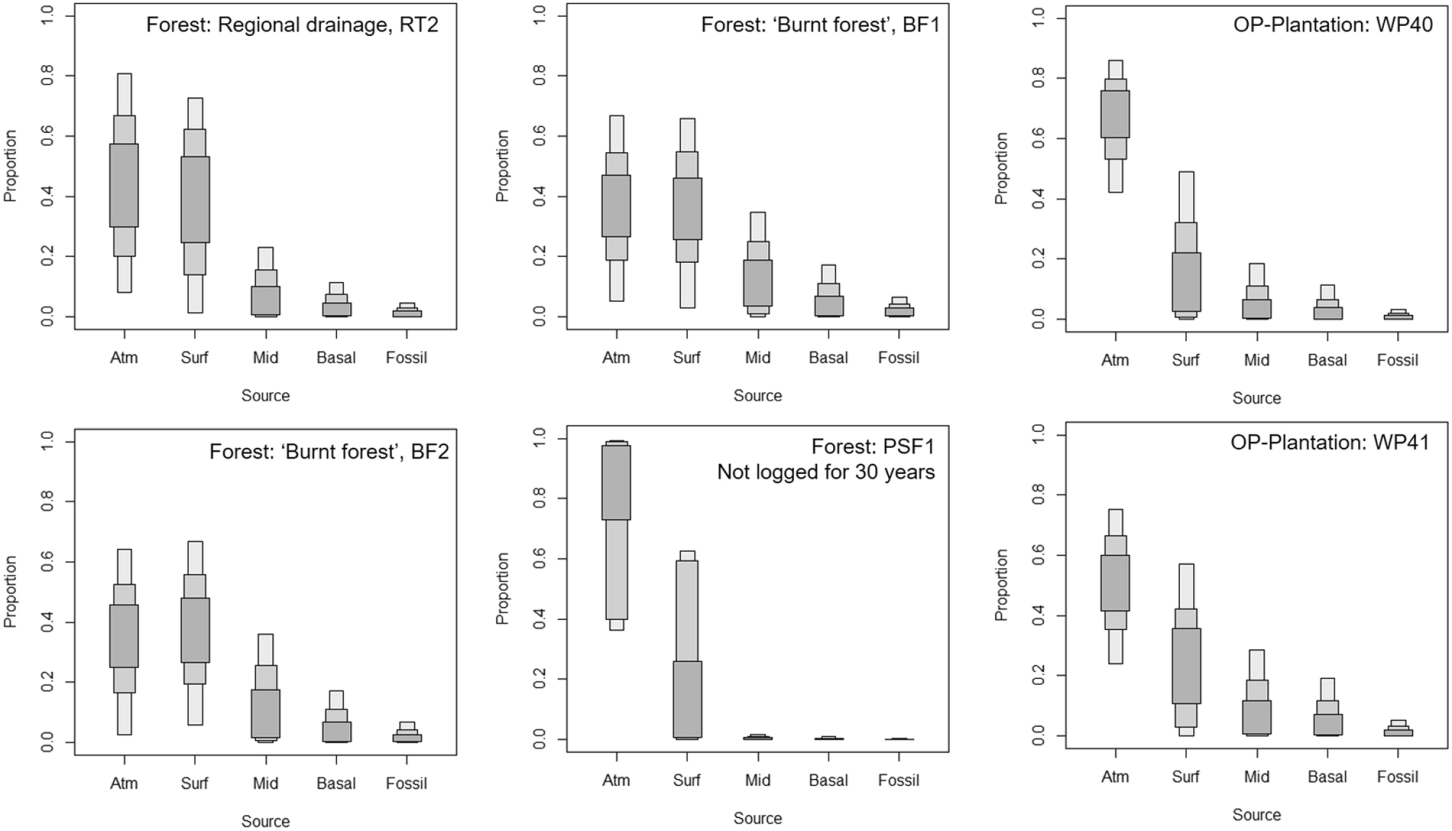
The outputs of the SIAR modeled contributions of different carbon age groups to forest (NSPSF) and OP-plantation (KLIA) CO_2_ efflux. The dark, light and lightest grey boxes represent respectively the 50%, 75% and 95% credible intervals of the estimates i.e. the contribution of a given source lies with this % probability in the interval. Although the same terminology has been used for the potential end members, these represent different age ranges and so this is a guide to where C may be derived rather than exactly the age of the carbon. For completeness, we have included a fossil C source in the modelling. If this was not present, the source contribution from mid- and deep-peat would be greater.

The SIAR analysis indicates that for all sites the CO_2_ efflux can come from a mix of all sources (Fig. 4).The modelled credible range contributions are broadly similar within and between the forest and the OP-plantation. However at OP-plantation WP40 the surface contribution is less than the atmospheric contribution, unlike the nearby WP41. The largest possible recently-fixed (atmospheric) C contribution is at forest-PSF1 (the logging moratorium site). The potential fossil contribution is small (<10% at all sites), particularly so for PSF1. Thereafter, the surface peat contributes more C to the efflux and the credible ranges are largest for this at forest-PSF1. PSF1 has smaller mid- and basal peat C contributions, these are more tightly-constrained than other sites, and are of similar magnitude to the fossil contribution. At all other sites potential mid- and basal contributions are much larger, but still less than atmospheric or surface credible ranges. Notably the surface peat age at OP-plantation sites (particularly WP41) is several hundred years old and therefore this contribution constitutes a loss of previously fixed C, whereas at the forest sites, surface peat is assigned a signature of being recently-fixed.

## Discussion

### Controls on dissolved organic and inorganic C

The [DOC] in all forest sites, other than the regional drainage system (RT2), is high e.g., 144.8 mg/l C at PSF1. Previously reported [DOC] concentrations for other drained peat swamp forests range from <6 mg/l C^13^ to 58 mg/l C^8^, although where [DOC] was unusually low, sulphuric acid leaching was invoked as a mechanism for suppressing DOC losses^13^.

[DOC] at OP-plantation WP40 was only 22.5 mg/l C and much less than nearby WP41, but within the range measured from palm oil drainage from the Malaysian province of Sarawak, northern Borneo (ranging from 8.3 and 82.5 mg/l C, but with most samples <60 mg/l C^14^). WP41 [DOC] (109 mg/l C) is greater than this Borneo drainage, and nearby palm oil sites on peninsular Malaysia (e.g. <13.3 mg/l C^13^). The decrease at WP40 could be due to dilution with a DOC-poor water, or UV-oxidation of organic material causing DOC loss (e.g.^15^). However, both OP-plantation sites had similar exposure and the drains were connected, thus it would seem unusual for UV-oxidation to significantly change [DOC] only in one site. Further, δ^13^C_DOM_ was not significantly enriched, so UV-oxidation seems unlikely to be the cause of the lower concentration. Addition of a [DOC]-depleted water seems more likely.

δ^13^C_DIC_ is typical of C_3_ vegetation (e.g.^1^) or is more ^13^C-enriched. The δ^13^C_DOC_ (Table 2) are more depleted than δ^13^C_DIC_ (Table 1), but this is unsurprising as there can be a kinetic fractionation ^13^C-enrichment in the DIC due to degassing^16^. The most ^13^C-enriched DIC sample was from Tengi River, which drains peat and areas of upland, non-peat forest, and may bring a groundwater contribution from limestone, present in the hills to the west^17^. However, groundwater is not dominant as SC in these systems is still low compared to typical karst drainage, where SC ranges from ~300 to 1100 μS/cm e.g.^18^.

δ^13^C_DIC_ at OP-plantation WP40 and WP42 were more ^13^C-enriched than at WP41 and all forest sites not on the main Tengi river (Table SI3). [Ca] were higher, consistent with a greater contribution of DIC from lime. However, ^14^C of the WP40 CO_2_ efflux was the youngest of the ‘disturbed’ sites (there are no data for WP42). Thus, the enriched δ^13^C_DIC_ at WP40 is not primarily attributable to an agricultural lime source as this would increase the age of the DIC pool and in turn, the CO_2_ efflux. The simplest explanation is differences in DIC loading within OP-plantation, and its efflux, cause an isotopic enrichment of the residual DIC pool.

At all other sampling locations, δ^13^C_DIC_ was very similar to that expected of soil CO_2_: assuming the δ^13^C_DOC_ is comparable to soil organic matter, then we would expect δ^13^C_DIC_ of approximately −25‰ and −23.5‰ for the forest and OP-plantation respectively. The ~2‰ ^13^C-enrichment in DOC in the OP-plantation than the forest may reflect ^13^C-enrichment of the remaining peat soils due to greater loss of a ^13^C-depleted pool (assuming a comparable starting composition), or it may be natural variation in the soil profile. The differing DOC ages between sites could reflect peat-depth sources and therefore vegetation differences. This is possible over short distances, for example, peat cores collected ~2 km apart from a pristine peat and a deforested peat dome had bulk δ^13^C_PEAT_ ranging from – 32.3 to −27.8‰ and −30.7 to −28.3‰, respectively^19^.

δ^13^C_DOC_ is internally consistent in the logged swamp forest and the oil palm plantation. However, δ^13^C_DIC_ showed intra-site differences, indicating these two C pools, although linked through respiration and UV-oxidation of DOC to DIC, can behave independently. DIC is influenced by loss (degassing) and mixing of multiple sources (soil respiration, DOC breakdown, groundwater – although the latter is less important in this context), whilst DOC behaves more conservatively.

### Dissolved and effluxed gas behavior

Efflux was smallest in the OP-plantation where water flowed very slowly. It was largest at forest-BF2, where the measurement was downstream of a small weir and flow was turbulent. Indeed, efflux on the same channel approximately 1 km upstream, at forest-BF1, was 2.00 ± 0.39 μmol C/m^2^/sec, considerably less than 23.99 ± 3.4 μmol C/m^2^/sec at forest-BF2. Both sites had similar [DIC] and pH (Table 1) and this difference thus reflects enhancing degassing by turbulent flow bringing gas to the surface and disrupting the surface boundary layer. Similarly, the greater efflux at forest-RT1 was due to faster water flow in the main stem river. Whilst the availability of dissolved CO_2_ is influential, the flow characteristics are a primary control, with the rate of efflux increasing as flow velocity increases^5^. Thus, caution is necessary in interpreting CO_2_ efflux without the context of local hydraulic properties.

Except for forest-BF2, the CO_2_ efflux rates are similar and at the lower end of the range observed in tropical peatland drainage channels in Indonesia (0.8–38.6 μmol C/m^2^/sec.^20^), from rivers in Sarawak draining a pristine peat dome (1.6–25.3 μmol C/m^2^/sec.^21^), and globally^5^. [DIC] is broadly similar for all sites (0.84–7.56 mg/l C) and there is not a consistent relationship between free CO_2_ calculated from pH and [DIC] and the rate of CO_2_ efflux. Thus, the concentration of DIC is not a primary control on CO_2_ efflux and low water velocity contributes to the low flux rates and explain inter-site variation.

It is likely in the wet season that the rate of degassing of CO_2_ from these systems would be higher when fluvial velocities are higher. However, a greater number of measurements spanning the dry and wet seasons are needed to provide a fuller C budget for whole system C losses, particularly in disturbed landscapes.

There are currently few measurements of CH_4_ concentrations in and flux from fluvial systems, but this is growing^22^. The concentrations measured here (0.5–247.5 μg CH_4_-C/l, Table SI3. Fig. SI1) are within the range observed in coastal swamps in Thailand (0.12–1297 μg CH_4_-C/l^23^) and in the wetlands of the Amazon basin (0.26–1297 μg CH_4_-C/l^24–26^). In both Thailand and the Amazon generally higher concentrations were measured during the wet season, and therefore higher dissolved [CH_4_] can be expected at our study sites in wetter conditions.

[CH_4_] was slightly greater in the clear-felled sites in both the forest and OP-plantation (Fig. 2; Table SI3) which may be due to reduced flow from smallscale drain blocks, clear-felling debris (forest-CF1), and in-growing reeds and grasses and patches of filamentous algae (forest-CF2). Alternatively the high C-loading in felled sites may have a greater respirative demand, such that methanogenesis may be more likely in the reduced oxygen concentration waters. Forest-PSF1 has not been logged for the past *c*. 30 years resulting in a more natural forest structure and closed canopy. Although the drainage channel is constructed, the surface water was algal-covered suggesting little water movement. The low [DO] (1.6%, Table 1) indicates stagnant water with little aeration. Therefore it is unsurprising that the highest CH_4_ concentration occured here. The reduced aeration from slow-moving drainage channels (infilling and further reducing flow) would promote low oxygen concentrations and support methanogenesis and prevent aerobic methane oxidation. As cleaning of drainage channels can decrease CH_4_ efflux^20^, the converse is likely true – CH_4_ can accumulate in drainage channels little disturbed.

The considerable surface algal growth would also increase surface tension and reduce gas diffusion - although this cover was punctuated by circles of open water, suggestive of methane ebullition (Fig. SI1). Thus CH_4_ concentrations may have been higher previously than measured. However when the algal cover is lost, perhaps by a storm blowing the material down-wind or as flow increases in the wet season, there could be a larger-scale episodic release of methane. There is a research need to explore fluvial [CH_4_] and controls on the significance of this C pool in the terrestrial-aquatic-atmospheric continuum, particularly where drainage channels are blocked on logged or developed peat swamps as restoration measures to increase C sequestration.

### Identifying the source of the effluxed C

Key to understanding the rate at which CO_2_ is returned to the atmosphere from terrestrial stores is to understand how much terrestrial C is being lost and of what age. This can be achieved by assessing rates of flux (e.g. the CO_2_ efflux here) and radiocarbon dating. However, tropical peatlands can be complex systems that develop in a stepwise fashion rather than continuously^27,28^ and so dating age ranges of source material where possible helps constrain the sources.

The basal ages of the two OP-plantation cores are very similar, ranging from 2446–2572 years BP with a mean age of 2508 years BP. The near-surface ages are also similar, ranging from 406 to 719 years BP, averaging years 562 BP. The basal ages at the forest site are older: 3861 and 3946 years BP. All basal ages are close to, but younger than when peat formation is considered to have commenced in Malaysia: approximately 5000 years ago controlled by sea level change^29^. However, this would have varied spatially and so these younger ages indicate peat formation started later, and at the forest site in North Selangor before the south Selangor peat (OP-plantation).

OP-plantation surface peats had pre-bomb ^14^C content, ranging in age from 406 to 719 cal. year BP. There are few measured ages of peat surface and basal ages in drained peatlands in peninsular Malaysia for comparison. The radiocarbon age of a peat sample obtained at a soil depth of 5 cm from Indonesian peatlands is similar at 890 ± 25 cal. years BP^30^. However elsewhere in SE Asia, peats have been reported to be younger, e.g. modern ^14^C concentrations were found at 82 cm depth in an Indonesian peat core^31^. Therefore, the older ages for OP-plantation peat may reflect peat loss.

Such loss would be unsurprising, for both sites, given that lowered water table supports organic matter respiration and there is likely fire loss of peat (see Fig. SI2). For these reasons we have used different SIAR mixing model scenarios to explore the partitioning of source contribution to the CO_2_ efflux, to understand whether the modern/fossil contribution is negligible, and if different depths of the soil profile gained different source importance.

The same end member ^14^C ages were used for all forest sites and the source contribution profiles SIAR generates are similar, except for PSF1, the site of logging moratorium. Here, gas efflux is more influenced by modern C (recently-fixed and surface peat derived) than elsewhere at the forest site. At all other forest sites the ^14^C content of effluxed CO_2_ was <100%modern (i.e. pre-modern; >0 years BP), and the SIAR analysis showed that the potential contribution from deeper layers of peat (mid and basal) was higher than at the site of logging moratorium, PSF1.The forest profiles (bar PSF1) are similar to the OP-plantation sites, although the age of CO_2_ efflux at OP-plantation WP40 was younger than the peat surface. SIAR demonstrates that a modern contribution is likely greater at OP-plantation WP40 than WP 41. This may be to do with site management e.g. more harvested palm debris on the peat surface acting as a source.

Thus, the ^14^C ages and SIAR source visualisation support our hypotheses – where disturbance had ceased the CO_2_ efflux predominantly reflects cycling of recently-fixed C. Further, this signal was apparent in the dry season where water tables should be lower and connection with older C stores, and so efflux, more likely. The young C efflux suggests the interpretation of recovery is robust. Conversely old CO_2_ constitutes efflux from drainage in sites subject to disturbance confirms land management drivers of atmospheric warming, and across a range of sites considered to have different levels of environmental harm.

The proportions of CO_2_ efflux derived from the DOC breakdown or from direct export from the soil were not measured. At all sites other than OP-plantation WP40, the age of the DOC was generally similar to the CO_2_ efflux (Fig. 3), indicating a source broadly the same, and thus DOC could be an important source of DIC and in turn CO_2_ efflux. The offsets in age between DOC-CO_2_ reflect process-related differences. At the forest site, CO_2_ efflux at BF1 and BF2 was slightly older than the DOC (the others are within measurement uncertainty). At OP-plantation WP40, the effluxed CO_2_ was relatively old, but with a ^14^C concentration suggesting it was younger than the DOC. The simplest interpretations for this are a contribution of old DIC at the forest site (from regional groundwater flow and /or soil respiration of aged organic matter), and young CO_2_ at the OP-plantation, from the atmospheric C fixed by the oil palms (suggesting a fossil C input to the DIC pool from agricultural lime is not strong). A modern contribution is unsurprising as the growth of oil palm indicates current C sequestration and so there will be modern C recycled. The difference in DOC-CO_2_ age efflux relationship between the OP-plantation two sites indicates intra-site differences in C cycling processes can occur and this needs to be considered when upscaling our understanding of C flow and losses.

Characterising the age of effluxed CO_2_ is not commonplace. There are few measurements of this component of efflux with which a comparison can be made. The only tropical forest site we can compare with is from the Peruvian Amazon^32^. In the Amazon sites, with the exception of an ephemeral stream in the rainforest (i.e. not connected to groundwater flow), old C contributed to CO_2_ efflux from drainage systems considerably different in size. These systems were in an area not subject to significant logging and land conversion and therefore old efflux was attributed to groundwater flow bringing in DIC from a geologically old source, or the remobilisation of OM in sedimentary terraces and from landslides.

DOC recovered from the Maludan River in Sarawak was modern indicating little peat degradation^21^, but this river drains an intact peat dome. DOC from two Malaysian oil palm plantation drainage channels in the dry season was dated to be 3184 and 4183 cal. years BP for abandoned and active plantations respectively^8^. This DOC is older than samples from the our OP-plantation and forest sites, but the location information^13^ shows these sites are closer to urban areas and main roads, and this may have introduced C of different ages to the drainage. The age of DOC from drained and degraded Borneo peat swamp forests ranged from modern to 1760 ± 268 cal. year BP^8^, which is still older than our forest site. Further, where aged DOC was being exported, it was older during the wet season, which is surprising as here the water table will be closer to the surface, and so a younger DOC contribution may be expected e.g. at five of six Indonesian oil palm drains where water table was <60 cm and maintained to be stable, post-1950 DOC-dominated oil palm drainage^33^. An older DOC pool may arise in the wet season from greater hydrological connectivity with older peat and flushing of a soil where the water table has been significantly lowered. However, as the wet season proceeds it may be after the initial flush of older C, C export from the upper soil profile takes place, and the DOC pool and so likely the CO_2_ efflux (Fig. 3), becomes younger. As the water table drops again in the dry season the age may become older. The length and duration of the dry season and how the water table depth is affected is therefore critical and should prime models of the peatland C cycling in the tropics.

### The significance of land use on the C cycle

Oil palm plantations are iconic as a symbol of tropical peatland degradation. However, the age of the oldest CO_2_ efflux from the forest reserve is comparable with the oldest plantation efflux (582 ± 37 vs. 567 ± 37 years BP, respectively) and in all cases, old gases came from recently-disturbed areas. Thus, conservation measures should not just focus on oil palm plantations, but drainage, burning, and clearing should be considered. The importance of drainage is apparent from a comparison of ^14^C_DOC_ of porewaters from two tropical forest peat domes in Brunei Darussalam, Borneo. Both sites had modern DOC^19^. However, unlike our forest site, the logged site was not drained, and the modern DOC in the porewaters, to depth, suggests a lack of drainage has mitigated peat loss and the C cycle may be broadly similar to the pristine site.

Restoring tropical swamp forests not yet converted to oil palm may be an ‘easier win’ than restoration of sites where the natural vegetation has been lost^9^. This was identified as a research priority for Malaysian peatlands^34^. Further, we need more secure understanding: turnover times have recently been identified as the key model inputs in terrestrial ecosystems^35^; knowing the speed of carbon cycling of different aquatic pools is equally as crucial as links from terrestrial to marine and atmospheric C reservoirs, and the sensitivities of each to environmental drivers of change are likely to differ.

Our data highlight that when old carbon is exported to fluvial systems, old CO_2_ is effluxed to the atmosphere through the terrestrial-aquatic-atmospheric C continuum. Previous research in more pristine tropical forests has shown old CO_2_ can be degassed due to a geologically dead C contribution^32^. However, the Malaysian field sites have been subject to land use change, and this activity is the strongest driver of old C in the system. The oxidation of organic matter, in peat deposited since ~4000 years BP, produces old DOC (although younger sources will contribute too) and this can be respired or oxidised to CO_2_. This DIC source is supplemented by leached soil DIC, which may be of a different age and younger - but as a smaller pool, the net CO_2_ efflux is still old. CH_4_ is present in surface waters, but it is not yet possible to comment on the significance of this pool size as so few [CH_4_] exist for fluvial systems. It is timely for this parameter to become a core measurement in terrestrial-aquatic-atmospheric C continuum research.

### Supporting effective landscape restoration, and carbon management and modelling

^14^C has proved to be an excellent tracer, both in confirming that old C is released to the atmosphere and in revealing that a drained and logged site can recover to have a C cycle more typical of an undisturbed site. Here, ^14^C measurement also showed that, although sites were disturbed, the drainage system is degassing C that has been recently-fixed, and this would not have been known if only surface peat ages were considered to represent the composition of resultant efflux as these would likely have the signal of peat loss. Elsewhere it has been shown that a modern C cycle still dominates, even if a tropical peat swamp has been logged, but not drained^19^. Thus, the application of ^14^C analysis/dating to understand terrestrial C loss arising from land use change, may be equally as valuable to reveal when a site has recovered and could act as a tool to assess the effectiveness of REDD or C-offset schemes that involve restoration. Given the link between old DOC and old CO_2_ efflux, the simplest approach for land managers may be to sample for ^14^C-DOC, which when appropriately sampled to avoid contamination and filtered and stored, retains integrity up to 3 months^36^, so suiting campaign fieldwork. This could be much simpler than paleoecological approaches e.g.^37^.

Focus on CO_2_ efflux is also needed. CO_2_ efflux ultimately derives from dissolved fluvial pools and so may be indirectly included in budgets of total C export if all dissolved C pools are being measured in export budget calculations. However, the longer the drainage channel the more time for reworking and degassing and just dissolved fluvial C estimates may miss efflux. Tall tower and aerial measurements of CO_2_ efflux are increasingly being used to calculate net ecosystem production, particularly in sensitive areas such as oil palm plantations and tropical forests. Here, apportioning net carbon loss/gain to the different components of the biome (e.g. oil palm, soils), may drive management policy, but will be inaccurate without quantifying the proportion of CO_2_ efflux that is from the drainage channel and is soil than plant respiration^2^. This may be possible by considering the surface footprint which the tower sees as wind direction changes, but in disturbed systems additional surface measurements of CO_2_ efflux for radiocarbon analysis would refine this understanding. Finally, CH_4_ should not be ignored in budgeting, or for assessing if C-cycling is dominated by modern C-flow.

This research contributes to a growing body of work that refines our understanding of the impact of anthropogenic activity on the immediate landscape (e.g.^8,13^) and of how we inform future projections of a landscape response to external drivers, be these short-term and anthropogenic (e.g. deforestation and drainage) or longer-term (e.g. climate feedback responses from such drainage). Larger ecosystem scale models^38–40^ lack age as a constraint on pool residence time or rate of C transfer. Carbon cycling models need to be revised to incorporate the rates of carbon flow and residence times, not just transfer between reservoirs. Without this we are neither identifying the significance of differing C contributions to atmospheric CO_2_ efflux, nor have the secure foundations to impose a driver of change.

## Materials and Methods

### Study area

Malaysia has the fourth highest area (25,889 km^2^) of tropical peat after Indonesia and the newly-identified peatland reserves of the Democratic republic of Congo and the Republic of the Congo^41^. However, following land use change, <5% of the intact peat swamp forests on Peninsular Malaysia remain and the rest are either highly-degraded or have been converted to oil palm agriculture^42^. In Malaysia there are now 57,380 km^2^ of palm oil plantations^9^.

Our two field sites were in Selangor state, which is approximately 810,000 ha in area and has the largest economy in Malaysia and a growing population of >5.5 million. The eastern part of the state is bordered by the Main Titiwangsa mountain range but descends into low hills and floodplains to the west. Peat soils dominate between the Selangor River in the north and Langat River in the south, and have formed over the past 5000 years, typically developing at a rate of between 2–5 mm/year^43^. Within the state peat covers 164,708 ha, with just over 81,000 ha of that maintained as forest reserves and the North Selangor Peat Swamp Forest (the ‘forest’ site) comprises the majority at 72,800 ha. Pilot monitoring has suggested the peats in the forest site are typically 3–6 m in depth, but the maximum depth recorded has been 10.15 m^44^. In South Selangor, where the oil palm plantation is, peats appear shallower, with a maximum depth of 2.2 metres observed within the study site.

In 1990, the State Authority gazetted the forest as a ‘reserve forest’ under the National Forestry Act (1984, amended 1992) and as a Class 1 Environmentally Sensitive Area. Prior to this status, the forests were classified as State land forests, and were subject to comparatively unrestricted logging since the 1930s. Consequently, *c*. 30% of the North Selangor PSF is categorised as highly-degraded (e.g.^45^). The complex history has resulted in a legacy of over 500 km of drainage canals dug to facilitate the transportation of timber (e.g.^46^).

Two major rivers drain the the forest site: the Bernam River entering from the north, and the Tengi River which traverses the swamp forests from east to west (Fig. 1). These are linked by an artificial canal (Fig. 1) constructed to meet the water demands of large-scale paddy rice production areas downstream, and to dilute the blackwaters from the forest swamp with the less-acidic Bernam River water. Sampling locations in the forest site were chosen to encompass a range of different land uses, from the main Tengi river channel carrying regional drainage from both peat and the adjacent uplands west of Tanjung Malim, composed of carboniferous marine shales, sandstones and limestones^17^, to localities of illicit burning (regular and non-regular), drained plantations and relatively intact secondary forest.

Since 1977 and especially since 2000, large areas of south Selangor peatlands have been converted to small holder agriculture, oil palm plantations, and most recently, the expansion of KLIA airport with the new terminal. The OP-plantation site was previously part of the wider South Selangor Peat Swamp Forest but now much of the oil palm plantation is entering its second generation. Here the landscape use was more homogenous than the forest site so only two sites were chosen (Table 1), both converted from forest in 2000 and representing the last areas on the 1^st^ cycle generation and the deeper peat areas of the plantation. OP-plantation WP40 (1.9–2.2 m) is on the southern edge of the plantation, with a drainage influence from a larger area of more recent conversion (2000 AD). Op-plantation WP41 (1.6–1.8 m peat depth) while still in 2000 plantings, is more proximate to second generation, older conversion planting in shallower peats. More details of the field sites and some images of the field sites are provided in the SI (Table SI1, Figs SI2, SI3).

### Sample collection and analysis

Over 2 years, we collected samples to determine C sources and identify the age of end members that may contribute to CO_2_ efflux. In July 2013, the dry season, fluvial samples were collected for the measurement of DOC, POC, DIC and CH_4_ concentration (concentration denoted by [*x*] e.g., [DOC]). Acid-washed Nalgene bottles were used to collect two 1-litre for [DOC] and [POC], and for [CH_4_]. Water chemistry parameters (pH, specific conductance (SC), dissolved oxygen (DO) and turbidity) were measured at each sampling point (YSI Pro Plus multi-parameter probe). Daily, on return from fieldwork, DOC samples were filtered through 0.7 μm pre-combusted glass fibre filters and the filtrate and filter papers were refrigerated until analysis (except during air freight to the UK). Immediate filtration and refrigeration supports storage for up to three months without compromising sample composition^36^.

δ^13^C_DIC_ and Δ^14^C_DOC_ were measured as these are key isotopic species to understand better fluvial C cycling (e.g.^32,47^). [DIC] and δ^13^C_DIC_ samples were collected by injecting 9 ml water sample into three pre-evacuated exetainers containing 150 μL of phosphoric acid to convert the DIC pool into CO_2_, preserving the sample and rendering it ready for headspace analysis^47^. The samples were refrigerated with inverted headspace until analysis. [DIC] and δ^13^C_DIC_ were measured contemporaneously from the same sample^47^ using CF-IRMS (Thermo-Fisher-Scientific Gas Bench/Delta V Plus at SUERC).

Prior to measurement of [DOC], samples were acidified to pH 3.9 with H_2_SO_4_ and sonicated to efflux inorganic carbon. [DOC] was measured using high-temperature, catalytic oxidation (Thermalox TOC 2020, Analytical Sciences). [POC] was quantified from the filter papers by loss on ignition (e.g.^48^).

Samples for measurement of dissolved [CH_4_] were analysed on the same day, except for the last set of samples from the forest site, which were analysed the following the day. Dissolved [CH_4_] was calculated from headspace measurement of ppm CH_4_ with a CH_4_ IR-detector (Detecto Pak-Infrared, DP-IR, HEATH) (^49^) and the dissolved CH_4_ calculated using a partition coefficient^50^. We transferred the collected water sample to a 1 L glass Kilner jar (Kilner, UK) fitted with connectors for headspace analysis. It is possible that some gas was lost during the transfer and so the concentrations should be considered minima.

The rate of surface water CO_2_ evasion was measured using a floating chamber connected to an infrared gas analyser (Li-840A, LI-COR). CO_2_ accumulation in the chamber headspace was measured over a four-minute period, three times at each sampling point and the fluxes calculated^50,51^. At key sites with different management history, the CO_2_ evaded into the floating chamber was collected for radiocarbon analysis by trapping in 13X Zeolite molecular sieve^52^. The chamber headspace was first scrubbed of atmospheric CO_2_ by pumping five chamber volumes (9.4 L) through a sodalime trap. After scrubbing, when the efflux accumulated in the chamber was of sufficient concentration for ^14^C measurement, the chamber headspace was pumped through the molecular sieve. After collection the tubing on the sieves was sealed. Thereafter, samples were stored at room temperature.

The DOC samples for radiocarbon analysis were collected in acid-washed Nalgene bottles. On return to the UK, DOC samples were acidified to pH 4, spurged with N_2_ gas, neutralised to just below pH7, rotary evaporated to a concentrate, freeze-dried to a powder and combusted. The molecular sieve trapped CO_2_ was released by heating^53^. For both sample types, sample CO_2_ was cryogenically purified and split into aliquots for δ^13^C (Thermo-Fisher-Scientific Delta V) and ^14^C measurement (reduction to graphite and accelerator mass spectrometry measurement at SUERC). As a quality control, distilled water samples were transported to Malaysia and filtered using the equipment used there and processed as per the field DOC samples. The results indicated no contamination by post-collection processing.

In June 2014 we recovered overlapping Russian cores from two sites at OP-plantation to radiocarbon date the surficial peats and the marine clay-peat transition to constrain the age range of soil C sources. One of these cores is from WP40, the same location CO_2_ efflux was measured, and the other core is from NE of WP40, at a site called KLIA 2010-2. The OP-plantation drains from approximately N to S and the second core location was chosen to sample peat in the upper site that was also second-generation oil palm plantation. Although of different core length, the base of the peat in both cores was identified by transgression into marine clay. Samples for radiocarbon analysis were selected from the surface and bottom of the peat deposits of KLIA-WP40 and KLIA-2010-2 at 3–8 cm and 181–183 cm, and 3–6 cm and 36–38 cm, respectively. Samples were prepared by sieving at 212 µm with the aid of deionised water and plant macrofossils were picked out under a microscope; where macrofossils were not abundant, bulk peat samples were measured.

It was not possible to collect cores from the forest sites at the time of sampling. However, cores from the forest were collected in 2017, very close to the peat swamp forest site (PSF1) - not logged for 30 years, and at a forest site converted to oil palm close to BF1 and BF2. Near-surface peats (3–8 cm depth) were dried and dated. This depth was chosen to avoid contamination of samples by modern (younger) root material that might penetrate the peat profile.

All samples underwent AMS ^14^C measurement at the Scottish Universities Environmental Research Centre. To account for mass dependent fractionation, following convention the ^14^C data were normalised to δ^13^C −25‰ and the results expressed as % modern and conventional radiocarbon age (years BP; where 0 BP = AD 1950^54^). Radiocarbon concentrations exceeding 100% modern cannot be assigned a conventional radiocarbon age and indicate the presence of post-bomb ^14^C and therefore a contribution from carbon fixed from the atmosphere post-AD1957 (when atmospheric ^14^CO_2_ first exceeded 100% modern^55^). In contrast, radiocarbon concentrations below 100% modern unambiguously indicate the presence of pre-bomb (“old”) carbon and can be assigned a conventional radiocarbon age.

Calcium concentration, [Ca], in drainage water filtrate was measured by AAS (Perkin Elmer Analyst 400) on return to the UK. [Ca] was used to assess if there was a limestone contribution to the C pool, either from regional drainage or in KLIA for the practise of liming the peat soils. The KLIA plantation owners confirmed that liming of the site does occur but did not in the areas we sampled in the year of sampling.

### Data analysis

To explore the relative importance of different C sources to the CO_2_ efflux we applied a Bayesian mixing model SIAR^56^ based on ^14^C ages using different end-members as % Enrichment Modern (to incorporate a post-bomb ^14^C contribution). We chose SIAR as it allows uncertainty in the end-members to be incorporated in the solution by identifying a range than using a fixed value. We did not use δ^13^C as degassed CO_2_ may be fractionated from δ^13^C_DIC_^57,16^ generating unrepresentative values for the sources of DIC. The SIAR approach allowed us to visualise how different sources may contribute to the CO_2_ efflux, and so compare between the forest and OP- plantation and between sites at these locations. The output gives credible intervals for the potential contribution of each source.

We were interested in where in the peat body C may be released that contributes to the CO_2_ efflux. Thus, we identified for the sites ages that represent the surface, mid-depth and bottom of the peat core. We have some dates for OP-plantation and for the forest swamp but not for all sites, nor all top and bottom. Thus, we allocated the following ages ranges (for ease summarised in Table SI.2).

At the OP-plantation we had top and bottom ages from two cores, one of which was sampled at the same site as WP40 was collected. Thus, for WP40 SIAR analysis the site-specific measured top and bottom core ages were used (Table 2). For WP41 the surface and basal ages were unknown and so the ages were estimated from pooling data from WP40 and an adjacent site, 2010-2. The mid-peat age was calculated from the age range generated for the basal and surface layer, ±0.47 (the largest SD in the ^14^C %modern estimate, equal to 38 years).

At the forest site, peat age data was more limited and so a common end-member composition was used for all sites based on the following. The basal peat age very close to PSF1, (not logged for 30 years), was 3861 BP, and at the oil palm converted site in the forest, close to the BF1 and BF2, was similar at 3946 BP. Thus, the mid-point of this range (including uncertainty) was used to represent the basal peat end member for all sampling locations. The surface peat was modern (102.13%modern) at a relatively undisturbed site within the reserve. The mid-peat age used was calculated the same way as for KLIA.

Fossil (^14^C dead) and recently-fixed ^14^C-end members were used across sites. The mean 2013 atmospheric CO_2_ composition of 102.76%modern^58^ was used to represent the recently-fixed fraction.

## Supplementary information

Supplementary Information
