# Supplementary material for: C mobilisation in disturbed tropical peat swamps: old DOC can fuel the fluvial efflux of old carbon dioxide, but site recovery can occur
Source: Sci Rep. 2019 Aug 7;9:11429. doi: 10.1038/s41598-019-46534-9 (PMC6685963; doi:10.1038/s41598-019-46534-9)

**Supplementary information for**

**C mobilisation in disturbed tropical peat swamps: old DOC can fuel the fluvial efflux of old carbon dioxide, but site recovery can occur.**

Susan Waldron^1^, Leena Vihermaa^1^, Stephanie Evers^2,3^, Mark H. Garnett^4^, Jason Newton^5^ _­_and Andrew C. G. Henderson^6^

Supplementary information.

Table SI1. Sample code, site description and co-ordinates of samples collected during the July 2013 campaign from North Selangor Peat Swamp Forest and an oil palm plantation near Kuala Lumpur International Airport. Sample coding commenced WP represents sites sampled and marked as a way-point with a GPS. Other coding for NSPSF are sites sampled more frequently as part of TROCARI field-research programmes. The location was not recorded for all sites.

| Sample | Site description | Northings | Eastings |
| --- | --- | --- | --- |
| North Selangor Peat Swamp Forest | | | |
| RT1 | River Tengi(RT)-Bernam canal that drains from the north | 03 59 116 | 101 34 983 |
| RT1B | Taken from a blackwater behind the bund that holds the canal | 03 35 463 | 101 24 974 |
| RT2 | River Tengi before joining the canal | 03 57 515 | 101 35 233 |
| RT3 | Side tributary of the R.Tengi with irregular burning (~ every 5 years) | 03 54 370 | 101 35 409 |
| RT4 | River Tengai that drains from the north | 03 53 780 | 101 32 329 |
| RT4B | Peat drain into the R.Tengai main river adjacent to RT4 |  |  |
| RT5 | River Tengai, just before meeting the main paddy canal | 03 47 851 | 101 22 726 |
| BF1 | Canal in a regularly-burnt forest (BF) area (annually for past 20 years; some young oil palm) | 03 42 878 | 101 33 670 |
| BF2 | The same canal as WP 25 but downstream of a drain block between WP24/5; ~ 1 km apart | 03 42 879 | 101 33 670 |
| PSF1 | Canal in a regenerated peat swamp forest (PSF), not logged for ~30 years, south of WP30 | 03 68 764 | 101 18 503 |
| PSF2 | Drainage canal at foot of observation tower and trek into regenerated forest, near road, palm oil to the north | 03 69 811 | 101 18 484 |
| OP1 | New road construction area (oil palm (OP) *approx 10-15yrs old) on NE, Forest on W | 03 66 966 | 101 24 425 |
| CF1 | Side drain in 2013 clear felled (CF) forest (5m upstream of a small drain block) | 03 66 230 | 101 32 843 |
| CF2 | Main drain in clear-felled 2013 area (roadside drain - draining larger CF and forest area, slow-moving) | 03 66 368 | 101 32 845 |
| Palm Oil Plantation near Kuala Lumpur International Airport | | | |
| WP40* | Canal draining palm oil plantation (southern edge of plantation) | 02 75 617 | 101 66 577 |
| WP41* | Canal draining palm oil plantation (to the N of WP 40) | 02 70 757 | 101 66 342 |
| WP42 | Canal draining palm oil plantation covered in algae | 02 72 053 | 101 64 520 |
| KLIACF1 | Drains in recently clear-felled oil palm area |  |  |
| KLIACF2 | Drains in recently clear-felled oil palm area | 02 46 878 | 101 39 895 |
| KLIACF3 | Drains in recently clear-felled oil palm area | 02 46 554 | 101 39 674 |

Table SI2. The scenarios used in the Bayesian End-Member Mixing Model SIAR for the peat source contribution. All sites are assumed to also have a fossil (^14^C dead) contribution (0 %modern) and a modern atm. contribution considered to be 102.76 ± 0.35. Where a surface or basal value was calculated from two cores, the full range (mean ± SD) was used to calculate the average, which accommodated differences in analytical uncertainty between cores influencing the age range. The intermediate ages were calculated from measured top and bottom ages, extended by the largest analytical uncertainty in the measured data to again give a range that was used in the model.

| Scenario | Surface peat | Intermediate | Basal peat |
| --- | --- | --- | --- |
| Forest  NSPSF  (all sites) | 102.12 ± 0.47  (After AD 1950)  Used surface ages from one core for both as only have this date | 81.75 ± 0.47  (1573 - 1665)  Used mid-value of the range of the two cores & range as uncertainty | 61.52 ± 0.61  (3823-3983 yr BP)  Used mid value of the range of the two cores & range as uncertainty |
| OP-Plantation | |  |  |
| WP40 | 94.73 ± 0.34  (406-464 yrBP)  Site-specific | 83.84 ± 0.47  (1371-1461 yr BP)  Estimated from the site-specific data | 72.87 ± 0.27  (2513 - 2572 yr BP)  Site-specific |
| WP41 | 93.26 ± 1.82  (405-719 yr BP)  Range of WP40 & adjacent core as similar and no site-specific ages | 82.12 ± 0.47  (1537-1629 yr BP)  Estimated from estimates | 73.17 ± 0.57  (2447 - 2572 yr BP)  Range of WP40 & adjacent core as similar and no site-specific ages |

Table SI3. Hydrochemical composition of the sites described in Table SI1. Not all parameters were measured due to time and sample bottle constraints (NM), particularly measurement of CO_2_ efflux which takes approximately 30 minutes. KLIA clear-felled sites had considerably higher specific conductivity than had been observed elsewhere and this raised concerns that chemicals had been used part of the clearing operations that could damage equipment and thus [DOC] was not measured. However, the CF sites been included given the importance of sharing data from palm oil plantations and particularly clear-felling.

| Sample | Date sampled | pH | SC | %DO | Temp˚C | [Ca] | [DIC] | δ^13^C-DIC | [DOC] | [POC] | CO_2_ efflux | [CH_4­_-C_aq_] |
| --- | --- | --- | --- | --- | --- | --- | --- | --- | --- | --- | --- | --- |
| Forest | |  |  |  |  |  |  |  |  |  |  |  |
| RT1 | 24/7/13 11.30 | 6.48 | 33.9 | 80.8 | 28.0 | 2.45 | 3.16 | -12.2 | 3.2 | 3.29 | 8.23 ± 0.53 | 4.5 |
| RT1B | 24/7/13 11.45 | 3.78 | 86.1 | 21.9 | 26.7 | 0.64 | 5.11 | -26.2 | 90.2 | 2.08 | NM | 17.9 |
| RT2 | 24/7/13 13.15 | 6.30 | 92.8 | 26.1 | 27.8 | 1.95 | 7.56 | -9.3 | 11.7 | 2.54 | 1.60 ± 0.07 | 2.5 |
| RT3 | 24/7/13 14.20 | 3.90 | 45.0 | 64.0 | 29.3 | 0.49 | 2.52 | -23.4 | 45.9 | 1.79 | 1.78 ± 0.42 | 6.6 |
| RT4 | 24/7/13 16.00 | 6.30 | 34.6 | 80.4 | 29.1 | 0.78 | 3.01 | -12.3 | 3.7 | 5.31 | 0.77 ± 0.35 | 4.9 |
| RT4B | 24/7/13 16.10 | 3.74 | 81.6 | 44.4 | 31.9 | 0.43 | 3.21 | -25.4 | NM | 1.39 | NM | 9.7 |
| RT5 | 24/7/13 18.45 | 6.25 | 34.3 | 68.5 | 29.5 | 0.72 | 3.23 | -13.1 | 5.4 | 2.96 | NM | 10.7 |
| BF1 | 25/7/13 11.05 | 3.74 | 75.2 | 6.7 | 29.7 | 0.96 | 5.40 | -25.0 | 83.6 | 0.94 | 2.00 ± 0.39 | 15.3 |
| BF2 | 25/7/13 14.55 | 3.71 | 74.7 | 19.3 | 28.9 | 0.95 | 6.00 | -25.7 | 83.0 | 0.76 | 23.99 ± 3.4 | 3.5 |
| PSF1 | 26/7/13 10.55 | 3.64 | 129.7 | 1.6 | 25.6 | 0.93 | 4.92 | -23.8 | 144.8 | 4.35 | 1.3 ± 0.04* | 247.5 |
| PSF2 | 26/7/13 15.00 | 3.58 | 90.3 | 30.6 | NM | 0.44 | NM | NM | 124.6 | 44.2 | NM | 6.7 |
| OP1 | 26/7/13 15.10 | 4.13 | 67.6 | 27.7 | NM | 0.32 | 3.05 | -25.0 | 127.5 | 3.62 | NM | 1.9 |
| CF1 | 26/7/13 15.25 | 3.14 | 72.7 | 23.1 | NM | 0.36 | 5.51 | -27.4 | 60.6 | 1.84 | NM | 8.6 |
| CF2 | 26/7/13 15.40 | 3.69 | 79.1 | 44.1 | NM | 0.22 | 4.74 | -23.1 | 71.9 | 8.60 | NM | 110.8 |
| OP-Plantation | |  |  |  |  |  |  |  |  |  |  |  |
| WP40 | 29/7/13 12.00 | 3.88 | 334.3 | 25.5 | 30.0 | 6.90 | 3.12 | -18.6 | 22.5 | 11.52 | 0.69 ± 0.11* | 0.5 |
| WP41 | 30/7/13 15.45 | 4.06 | 206.6 | 54.6 | 27.7 | 2.91 | 0.84 | -24.5 | 109.0 | 12.07 | 0.51 ± 0.08* | 1.4 |
| WP42 | 30/7/13 14.30 | 3.89 | 168.0 | 18.8 | 33.2 | NM | 3.62 | -20.6 | 153.4 | 24.71 | NM | 8.3 |
| KLIACF1 | 30/7/13 13.30 | 2.14 | 1359 | 9.0 | 32.1 | 1.98 | NM | NM | NM | NM | NM | 28.5 |
| KLIACF2 | 30/7/13 13.35 | 2.65 | 2353 | 23.3 | 29.8 | 0.21 | NM | NM | NM | NM | NM | Not detected (0.0) |
| KLIACF3 | 30/7/13 13.40 | 2.87 | 1783 | 22.1 | 31.3 | 0.38 | NM | NM | NM | NM | NM | 14.1 |

Table SI4. Site categorization for the [CH_4_-aq] land use analysis in Figure 2:

| Sample | [CH_4­_-C_aq_] | Categorisation |  |
| --- | --- | --- | --- |
| Forest |  |  |  |
| RT1 | 4.5 | Regional drainage |  |
| RT1B | 17.9 | Within site artificial drainage |  |
| RT2 | 2.5 | Regional drainage |  |
| RT3 | 6.6 | Within site artificial drainage |  |
| RT4 | 4.9 | Regional drainage |  |
| RT4B | 9.7 | Within site artificial drainage |  |
| RT5 | 10.7 | Regional drainage |  |
| BF1 | 15.3 | Within site artificial drainage |  |
| BF2 | 3.5 | Within site artificial drainage |  |
| PSF1 | 247.5 | Forest not logged for 30 years |  |
| PSF2 | 6.7 | Within site artificial drainage |  |
| OP1 | 1.9 | Site boundaries |  |
| CF1 | 8.6 | Drainage in felled areas |  |
| CF2 | 110.8 | Drainage in felled areas |  |
| Oil Palm plantation |  |  |  |
| WP40 | 0.5 | Site boundaries |  |
| WP41 | 1.4 | Within site artificial drainage |  |
| WP42 | 8.3 | Within site artificial drainage |  |
| KLIACF1 | 28.5 | Drainage in felled areas |  |
| KLIACF2 | Not detected (0.0) | Drainage in felled areas |  |
| KLIACF3 | 14.1 | Drainage in felled areas |  |

Fig. SI1. Box and whisker plot of the [CH_4_-aq] as a function of site sampled -the forest reserve (NSPSF) or the OP-plantation – showing the maximum, minimum, median and 1^st^ and 3^rd^ quartiles. Outliers are shown and the forest site has more outliers to the population composition.


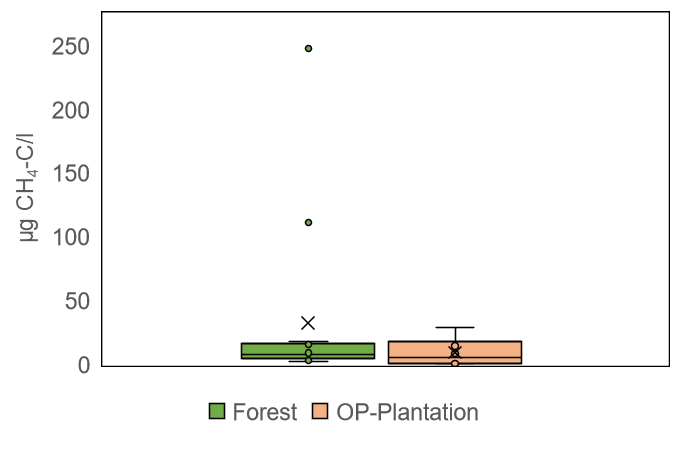


Fig.SI2 The algal-covered surface of the drainage system at PSF1 in Forest reserve, with the algal-surface cover punctuated by rounded breaks that could have been caused by the upward pressure of ebullitive methane fluxes e.g. in front of the log in the water to the left-hand side of the image are two larger circular holes but there are many smaller ones.


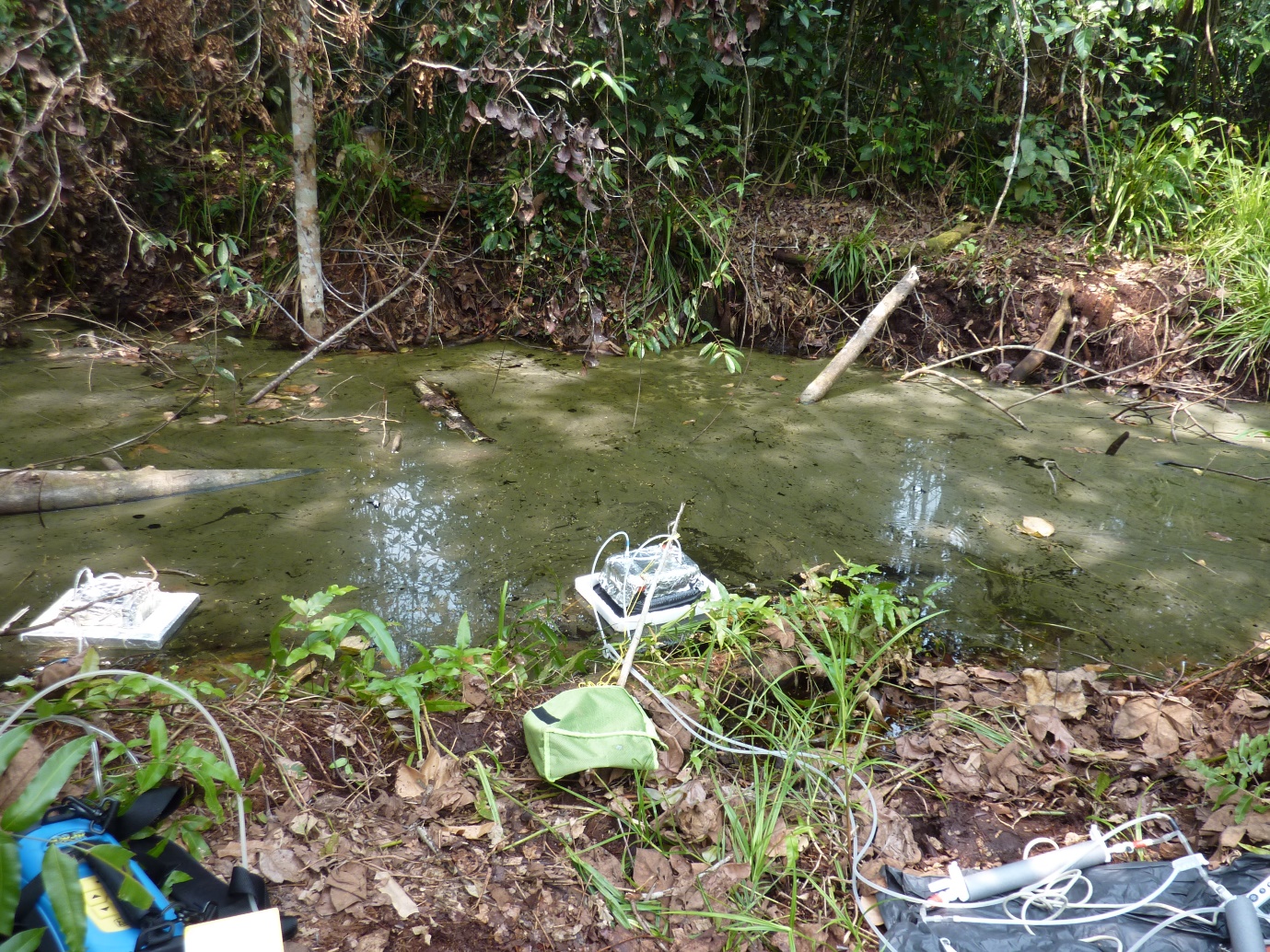


Fig. SI3 An example of the peat surface at the OP-Plantation, where blackened showing signs of fire possibly smouldering underground.


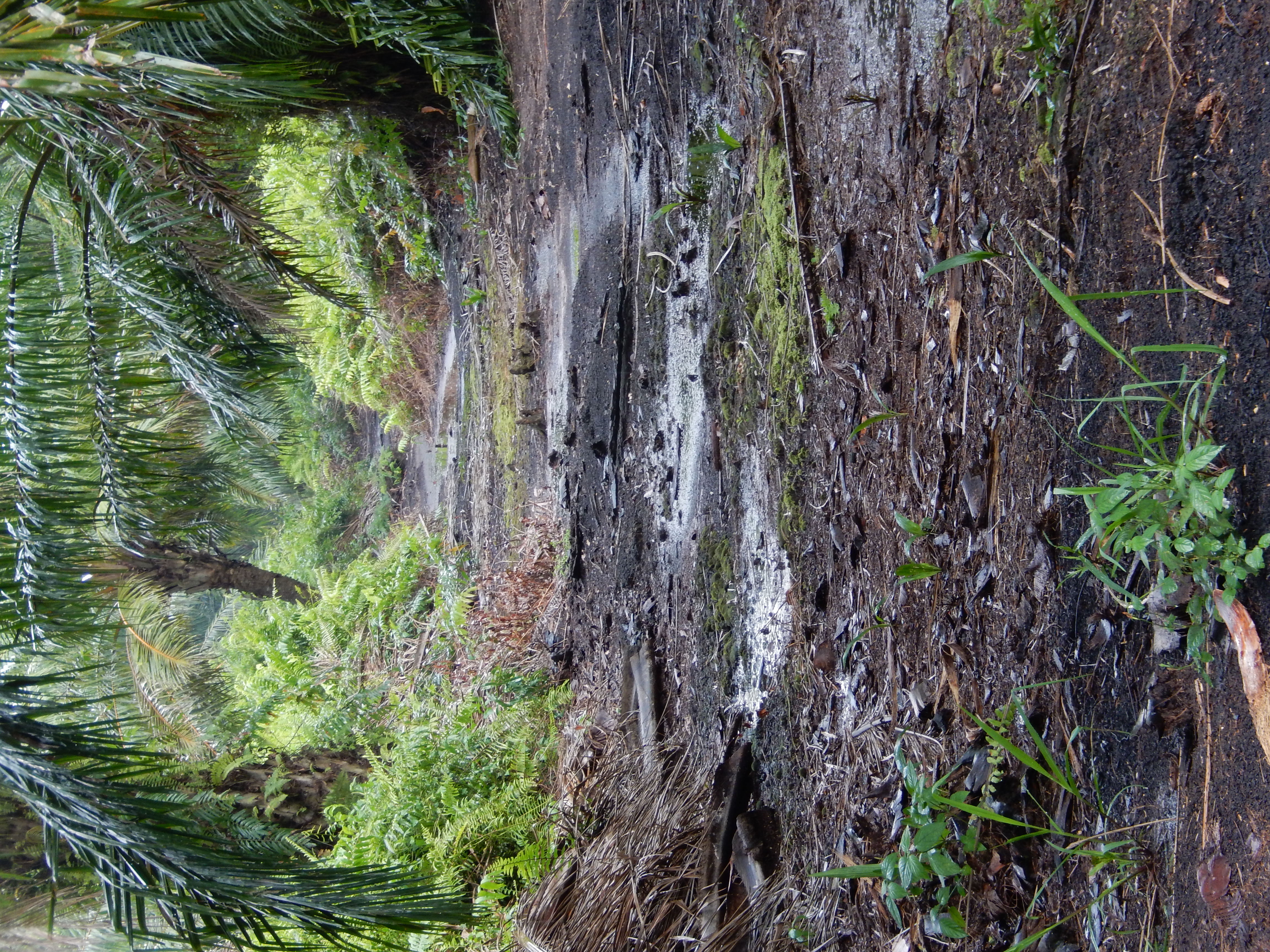

Supplement: Supplementary file 1 — Supplementary Information [file 41598_2019_46534_MOESM1_ESM.docx]
